# Assessment of Potential Drug–Drug Interactions of Psycholeptics and Antidepressants in Outpatient Settings †

**DOI:** 10.3390/pharmacy12060174

**Published:** 2024-11-22

**Authors:** Iva Marović, Ivana Marinović, Vesna Bačić Vrca, Ivana Samardžić

**Affiliations:** 1 Department of Clinical Pharmacy, University Hospital Dubrava, 10 000 Zagreb, Croatia; 2 Faculty of Pharmacy and Biochemistry, University of Zagreb, 10 000 Zagreb, Croatia

**Keywords:** mental health, drug–drug interaction, psychotropic drugs, outpatients, pharmacist

## Abstract

Mental health is an important segment in preserving overall health and represents a significant public health issue. In modern times, mental health disorders have risen, often requiring complex pharmacotherapy and chronic monitoring. The aim of this research was to determine the prevalence and clinical significance of potential psychotropic drug interactions in outpatient settings and compare the differences in potential drug–drug interaction (pDDIs) exposure with age. The psychotropic drugs included antipsychotics—N05A, anxiolytics—N05B, hypnotics and sedatives—N05C, and antidepressants—N06A. This retrospective study analyzed prescribed pharmacotherapy in 492 outpatients who were treated with at least one psychotropic drug. We determined 1.64 prescribed psychotropic drugs per patient and 2.2 pDDIs that involved psychotropic drugs. In total, 2285 pDDIs were recorded, of which almost half (47.6%) were pDDIs with psychotropic drugs. More prescribed psychotropic drugs were found in patients younger than 65 years, and equal exposure to pDDIs of psychotropic drugs (*p* = 0.5077) was found in both age groups. The most commonly identified psychotropics involved in pDDIs were benzodiazepines, promazine, and zolpidem. The results indicate that psychotropic drug interactions represent important drug-related problems for primary health care. The widespread use of psychotropic drugs and the determined clinical significance of their interactions require pharmacist interventions which can reduce the prevalence of pDDIs and increase patient safety.

## 1. Introduction

Mental health represents a significant public health issue, and it is an important segment in preserving overall health. Mental disorders have a relatively high prevalence and often have a chronic course [1]. Psychotropic drugs are regularly featured in annual drug consumption reports as one of the most utilized medications [2,3]. Mental disorders constitute a significant cause of unproductivity, disability, and significantly decreased quality of life [4]. Contemporary times register an increase in mental disorders, often requiring complex pharmacotherapy and chronic monitoring.

Even though psychotropic drugs are rapidly evolving, their pharmacological profile is often associated with different drug-related problems [5,6,7,8]. The most common problems related to psychotropic drugs include determining the appropriate dose, duration of drug use, implementation of deprescription protocols, side effects, adherence, and drug–drug interactions (DDIs). Therefore, to enhance their effectiveness and safety, the use of psychotropic drugs requires regular evaluation in all aspects of their safe use.

DDIs result in a modification of the action of one or more concurrently administered drugs, which can then result in decreased or increased drug effect, treatment failure, or increased drug toxicity [9,10]. Sometimes concurrent psychotropic drug use represents target DDIs, and they are used to achieve better and faster treatment outcomes. However, the synergistic interactions of psychotropic drugs should also be closely monitored because they may be associated with an increased risk of adverse drug reactions.

Certain groups of patients are at an increased risk of developing actual drug interactions [11]. The most sensitive among them are elderly patients. Due to numerous aging-related physiological changes, elderly patients are particularly vulnerable to the use of psychotropic drugs [12]. However, it should be emphasized that the use of psychotropic drugs is becoming increasingly common in younger patients as well [13,14]. A larger number of studies analyze the occurrence of DDIs in hospitalized patients, while there is less available data about DDIs in outpatient settings [15,16].

The aim of this research was to determine the prevalence and clinical significance of psychotropic potential DDIs (pDDIs) in outpatient settings and compare the differences in pDDI exposure regarding age.

## 2. Materials and Methods

A retrospective analysis included prescribed pharmacotherapy of outpatients. Data collection and analysis were conducted over a period of one year. Only patients prescribed a minimum of two medications, where at least one of which belonged to the observed groups, were included in the study. The observed psychotropic drugs classified according to the Anatomical Therapeutic Chemical (ATC) classification system were antipsychotics (N05A), anxiolytics (N05B), hypnotics and sedatives (N05C), and antidepressants (N06A). The International Classification of Diseases (ICD) was used for disease classification. Over-the-counter medications were not included in this study due to the unavailability of an official registry of non-prescription drugs in Croatian community pharmacies.

This study included equal samples of pharmacotherapies from ten community pharmacies across Croatia. The analysis was conducted based on information recorded in patients’ pharmacotherapy cards. Besides the dispensed drug, a pharmacotherapy card records the indication for which the drug was prescribed, dosage, quantity dispensed, the route of drug administration, and recommendation for drug intake.

*Lexi-interact* (an online interaction checker, provided from UpToDate, 2023) was used for analyzing pDDIs. The *Lexi-interact* program categorizes DDIs based on the degree of clinical significance into five categories: A (interaction has no clinical significance), B (intervention not necessary, insufficient data available), C (increased monitoring required, intervention as needed), D (therapy intervention necessary), and X (contraindicated use). Only categories C, D, and X were considered clinically significant. Lexicomp has a sensitivity of 97% and a specificity of 90% [17]. The results were also categorized according to age groups:

- younger than 65 years;
- 65 years and older.

Microsoft Excel Office 2010 and GraphPad Prism 8 software (version 8.01, GraphPad Software) were used in data processing. The Shapiro–Wilk test was used to test the normality of distribution, and the Mann–Whitney test was used for analyzing differences. Tests were considered statistically significant if *p* < 0.05.

## 3. Results

This research included 492 patients, and the average age was 67.7 years (Table 1). A total of 3097 drugs were identified, of which 809 (26.1%) were psychotropic drugs (psycholeptics and antidepressants). On average, each patient had 6.3 medications, with an average of 1.6 psychotropic drugs per patient. The total number of diagnoses was 1996, with an average of 4.1 diagnoses per patient. A total of 2285 potential clinically significant DDIs were determined, in which almost half of the pDDIs (47.6%) were identified to involve psychotropics. The average number of pDDIs of psycholeptics and antidepressants per patient was 2.2.

Table 2 presents the most commonly prescribed psychotropic drugs. Anxiolytics and hypnotics were the most frequently prescribed medications among the observed medication classes of drugs (50.2%). Table 2 shows the most commonly prescribed drugs. The most frequently prescribed anxiolytic was diazepam, hypnotic was zolpidem, antidepressant escitalopram, and antipsychotic promazine.

The most common indications for the use of psycholeptics and antidepressants are presented in Table 3. Anxiety and sleep disorders were the most common indications for psychotropic drug prescription.

Non-elderly patients showed a higher number of psychotropics per patient (1.8 vs. 1.6, *p* = 0.0275), while elderly patients showed higher number of prescribed total drugs, other drugs, and diagnoses (*p* < 0.0001). Table 4 shows a detailed comparison of the number of drugs and diagnoses according to age.

A statistically significantly higher number of total pDDIs were determined in patients ≥ 65 years. DDIs of category C and D were more prevalent among the elderly, but no difference was noted in category X between the two age groups (Table 5).

Table 6 presents differences in the pDDIs of psycholeptics and antidepressants between younger and older patients. There was no statistically significant difference in the number of pDDIs with psychotropic drugs among the observed age groups. A statistically significant difference was not identified among different DDI categories considering the defined age groups.

Table 7 summarizes the most frequent pDDIs involving psychotropic drugs, with benzodiazepines being the most represented. The most common potential DDI consequence was the increased risk of central nervous system (CNS) depression. Also among the most frequent consequences was an increased risk of QTc prolongation and the possibility for serotonin syndrome occurrence. The most frequent pDDI in both the observed age groups was interaction between benzodiazepine and tramadol. The most common type X interaction was between promazine and furosemide, which can result in QTc-prolongation.

## 4. Discussion

DDIs can complicate the course of treatment and jeopardize patient safety [18,19]. Mental disorders are often present with other chronic illnesses. Polypharmacy increases the risk of DDI occurrence [20]. The treatment of mental disorders usually requires use of more than one drug, which is often the case for other chronic diseases as well [21]. Multimorbidity is more present in the elderly, but also increases at younger ages [22,23,24]. In our study, one-third of the prescribed medications were psychotropic drugs. The consumption of these drugs is on the rise, with particular concern about their increasing usage among younger patients.

Therefore, the aim of this study was to identify potential clinically significant DDIs of psychotropic drugs and to determine differences in DDI exposure in both age groups. The average age of the patients was 67.7 years. Most countries have adopted the concept of 65+ as the threshold for the elderly population. In the European Union in 2022, the share of older individuals was already over one fifth of the total population (21.3%) [25,26].

In this research, there was a higher percentage of women, 71.1%. This finding is in accordance with the data from previous studies that show a greater inclination among women to use psychotropic drugs. Studies have consistently shown that women more frequently suffer and report mental disorders. Research from Boyd et al. was conducted in 10 European countries and evaluated a 12-month prevalence of psychotropic drug use. In Portugal, it was found that 30.4% of women were taking psychotropic medications, and the highest percentage of male population using these drugs was recorded in Belgium with a share of 12.8% [27].

It is estimated that 970 million people worldwide suffered from some form of mental disorder in 2019, with anxiety disorders being the most prevalent [1]. In our study, the most common indications for psychotropic drug prescription were anxiety disorders (F41, other anxiety disorders and F41.2, mixed anxiety and depressive disorder). Anxiety disorders often co-occur with other mental disorders [28,29]. Anxiety disorders usually precede other mental disorders, which imposes the need for the timely recognition of anxiety disorders as an opportunity for preventing the introduction of other psychotropic drugs.

Epidemiological studies indicate a higher prevalence of anxiety disorders among patients younger than 50 years old. After the age of 50, the occurrence of anxiety disorders significantly decreases. An epidemiological study conducted in Europe revealed that mental disorders have a higher 12-month prevalence in younger patients compared to those older than 65 years. The highest prevalence was recorded in the 18–24 age group (16.5%), followed by patients aged between 35 and 49 years (12.4%) [30,31]. Research published in 2021 showed that the highest percentage of adults who received any form of mental health therapy was in the 18–44 age group (23.2%), followed by adults aged 45 to 64 (21.2%) and those aged 65 and older (18.9%) [32]. The aforementioned data are consistent with our results and may explain why a high consumption of psychotropic medications was observed among younger patients in this study, which consequently contributed to a higher number of psychotropic drug interactions.

Overall, we determined 2285 pDDIs, of which significant portion (47.6%) were psychotropic drug interactions. Among psychotropic drug interactions, 64.9% were category C, 32.2% were category D, and 2.9% were category X DDIs. On average, there were 2.2 interactions involving psychotropic medications per patient. Considering all the determined drug interactions, older patients had a larger number of pDDIs than younger patients (*p* = 0.0095), but it should be noted that the results did not show a statistically significant difference in the number of psychotropic drug interactions between older and younger patients (*p* = 0.5077).

Benzodiazepines were the most represented in psychotropic drug interactions. Research imposes that the use of benzodiazepines is often prolonged [5]. According to recommendations, benzodiazepine therapy should be as short as possible, using the lowest effective dose. The recommended upper limit for the duration of benzodiazepine therapy, which includes a tapering-off period for the gradual cessation of treatment, is 12 weeks [33]. As this study suggests, the regular evaluation of benzodiazepine interaction risk is necessary for both patient groups. Benzodiazepines with a long half time (diazepam, flurazepam) are not recommended for elderly patients [34]. The most common D interaction was between benzodiazepines and tramadol in both age groups, in the elderly with alprazolam and in younger patients with diazepam. The half-life of diazepam is 90 h and alprazolam has a t_1/2_ of 8–12 h. Patients should be warned of slowed or difficult breathing and/or sedation and other related signs and symptoms of CNS depression if it is not possible to avoid this drug combination [35,36]. The concurrent use of tramadol with other drugs that cause depression of the CNS requires increased monitoring. Deprescribing benzodiazepines can significantly impact the occurrence of psychotropic drug interactions. The discontinuation of benzodiazepines should be gradual and official protocols for deprescribing should be followed [37,38,39]. Pharmacist counseling for patients about the risks of benzodiazepine use and about proper drug administration can significantly influence the rationalization of benzodiazepine use [5,40].

In this study, the most common potential consequence of psychotropic drug interactions was an increased risk of CNS depression. The mechanism of these interactions is that CNS depressants can enhance the depressant effect of opioids or other CNS-depressing medications. Benzodiazepines and zolpidem are positive allosteric modulators that enhance the response to Gamma-Aminobutyric Acid (GABA). GABA is an inhibitory neurotransmitter. It reduces a nerve cell’s ability to receive, create, or send chemical messages to other nerve cells. Tramadol is an opioid analgesic with central action. It is a non-selective μ, δ, and κ agonist of opioid receptors with a higher affinity for the μ receptor. Other mechanisms that contribute to the analgesic effect are the inhibition of the re-storage of noradrenaline and the stimulation of the release of serotonin. GABA receptors induced by tramadol have been shown to be secondary to its opioid receptor agonist activity [41].

The concomitant use of two or more agents that possess an ability to enhance central serotonergic activity may increase the risk for serotonin syndrome, a condition of serotonergic overstimulation characterized by autonomic, neuromuscular, and neurologic effects. Serotonin syndrome is characterized by hypertension, tachycardia, tachypnea, hyperthermia, hyperreflexia, hypertonia, and hypersalivation [42,43,44]. The onset of symptoms is usually within 12 h of the initiation of treatment. The mechanism of development involves an excessive accumulation of serotonin, which can occur due to the inhibition of serotonin uptake, reduced serotonin metabolism, increased serotonin synthesis, enhanced serotonin release, and activation of serotonin receptors.

The most common potential consequence of X psychotropic drugs interactions was an increased risk of prolonging the QTc interval. Prolongation of the QTc interval is associated with an increased risk of ventricular tachycardia *Torsades de pointes* (TdP). Antipsychotics and antidepressants have the risk of prolonging the QTc interval as a side effect. Systematic review and meta-analysis of the included studies determined that QT prolongation DDIs in psychiatric patients was found to be 42% [45]. Therefore, it is necessary to assess the risk-to-benefit ratio of psychotropic drugs and identify other risk factors and potential for interference with other drugs with QTc prolongation [46,47]. Risk factors for prolonging QTc are bradycardia, hypertension, advance age, female gender, electrolyte disturbances, presence of heart disease, and diabetes [48,49,50]. Today, various tools and algorithms have been developed for predicting the risk of QTc prolongation/TdP. Algorithms suggest correcting modifiable risk factors such as electrolyte disturbances (hypokalemia, hypomagnesaemia) prior to starting therapy. In accordance with the estimated prolongation of the QTc interval, a risk level monitoring plan should be implemented. Monitoring should include observation of ECG and QTc prolongation symptoms. Symptoms include palpations, lightheadedness, and dizziness and should be monitored. Depending on the present factors, an initial ECG should be performed and then repeated after achieving steady-state levels. In patients with congenital long QTc syndrome, an alternative drug should be considered if a drug with a high risk of QTc prolongation is prescribed [51]. Other medications that can prolong QTc interval include antiarrhythmics, antimicrobials, antihistamines, and antiemetics.

Pharmacotherapy management of psychotropic drugs is a challenging process regarding effectiveness and safety, and it should also include DDI evaluation. This research shows that psychotropic drug interactions are an important drug-related problem in primary health care for the elderly and younger patients. A proactive pharmacist involvement in psychotropic drug interaction surveillance and proper interventions can contribute to safer and more rational use of psychotropic drugs. In cooperation with other health care professionals, pharmacists should contribute to prescribing safer drug combinations and providing patients counseling regarding DDIs.

The literature indicates that it is also necessary to introduce some new possibilities in the prediction of drug interactions. Certain recent papers suggest that artificial intelligence could also contribute to the prediction of drug interactions [52,53]. However, expert professional interpretation in assessing the benefit–risk ratio of identified pDDIs is necessary. In order to make the best possible interventions, patient s’ clinical status and clinical treatment priorities must be evaluated.

There are several limitations to this research. This study only provided results for DDI exposure in outpatient settings. In hospital settings, a different profile of drugs can be used, and the occurrence and types of interactions can differ. This method of retrospective analysis cannot evaluate non-prescription drug use in the study patients. A database of non-prescription drugs is not available and is not mandatory in community pharmacies. Non-prescription drugs usually have a lower potential for drug interactions, but their inclusion in analysis could also contribute to certain conclusions. The inclusion of laboratory findings, for example, parameters of kidney and liver function, would additionally help in assessing the risk of actual drug interaction occurrences. Increased exposure to the drug due to impaired liver and kidney function can significantly increase the risk of actual drug interactions.

## 5. Conclusions

Potential psychotropic drug DDIs comprise a significant share of determined potential drug interactions in outpatients. Even though older patients had a larger number of total pDDIs than younger patients, no difference in the incidence of psychotropic drug interactions was found between these two patient age groups. The most common consequences of the determined potential psychotropic drug interactions were increased risk of CNS depression and risk of prolonging QTc interval. Psychotropic drug interactions represent an important drug-related problem for primary health care and require more active pharmacist involvement.

## Figures and Tables

**Table 1:** Patients characteristics.

Characteristics	Sample (n = 492)
Age, years, average (range)	67.7 (20–95)
Gender, women, n (%)	350 (71.1)
Total number of drugs	3097
Average number of drugs (range)	6.3 (2–18)
Total number of psycholeptics and antidepressants	809
Average number of psycholeptics and antidepressants (range)	1.6 (1–6)
Total number of diagnoses	1996
Average number of diagnoses (range)	4.1 (1–11)
Total number of pDDIs	2285
Average number of pDDIs (range)	4.6 (0–30)
Total number of pDDIs of psycholeptics and antidepressants	1088
Average number of pDDIs of psycholeptics and antidepressants (range)	2.2 (0–21)

pDDIs—potential drug-drug interactions.

**Table 2:** Most commonly prescribed drugs from observed groups.

Medication	n (%)
**Antipsychotics**	119 (14.7)
Promazine (N05AA03)	30 (3.7)
Quetiapine (N05AH04)	18 (2.2)
Sulpiride (N05AL01)	18 (2.2)
Haloperidol (N05AD01)	10 (1.2)
Risperidone (N05AX08)	9 (1.1)
Olanzapine (N05AH03)	9 (1.1)
Aripiprazole (N05AX12)	8 (1.0)
**Anxiolytics**	406 (50.2)
Diazepam (N05BA01)	150 (18.5)
Alprazolam (N05BA12)	140 (17.3)
Oxazepam (N05BA04)	79 (9.8)
Lorazepam (N05BA06)	31 (3.8)
Bromazepam (N05BA08)	6 (0.7)
**Hypnotics and sedatives**	121 (15.0)
Zolpidem (N05CF02)	87 (10.8)
Nitrazepam (N05CD02)	34 (4.2)
**Antidepressants**	163 (20.1)
Escitalopram (N06AB10)	37 (4.6)
Sertraline (N06AB06)	24 (3.0)
Mirtazapine (N06AX11)	20 (2.5)
Paroxetine (N06AB05)	18 (2.2)
Tianeptine (N06AX14)	13 (1.6)

**Table 3:** Most common indications for the use of psycholeptics and antidepressants.

ICD-10-CM Diagnosis Code		n (%)
F41	Other anxiety disorders	82 (16.7)
F51	Sleep disorders not due to a substance or known physiological condition	74 (15.0)
F41.2	Mixed anxiety and depressive disorder	64 (13.0)
F32	Depressive episode	58 (11.8)
F06	Other mental disorders due to known physiological condition	44 (8.9)
F20	Schizophrenia	32 (6.5)
F06.3	Mood disorder due to known physiological condition	24 (4.9)
F43.1	Post-traumatic stress disorder (PTSD)	16 (3.3)
F43.2	Adjustment disorders	15 (3.0)
F06.2	Psychotic disorder with delusions due to known physiological condition	13 (2.6)

ICD-10-CM—International Classification of Diseases, Tenth Revision, Clinical Modification.

**Table 4:** Differences in the number of drugs and diagnoses according to age.

	≥65 Years	<65 Years	*p*
**Patients, n**	302	190	
**Total number of drugs, n**	2099	998	<0.0001
**average ± SD**	7.0 ± 2.9	5.3 ± 2.3	
**range**	2–18	2–11	
**Number of psycholeptics and antidepressants, n**	474	335	0.0275
**average ± SD**	1.6 ± 0.9	1.8 ± 1.0	
**range**	1–5	1–6	
**Number of other drugs, n**	1625	663	<0.0001
**average ± SD**	5.4 ± 2.8	3.5 ± 2.2	
**range**	0–14	0–9	
**Number of diagnoses, n**	1348	648	<0.0001
**average ± SD**	4.5 ± 1.9	3.4 ± 1.7	
**range**	1–11	1–9	

SD—standard deviation.

**Table 5:** Potential DDIs of all drugs according to clinical significance and age.

	≥65 Years	<65 Years	*p*
**Patients, n**	302	190	
**pDDIs, n (median)**	1553 (4.0)	732 (2.5)	0.0095
**average rank**	259.6	225.7	
**range**	0–30	0–24	
**Category C pDDIs, n (median)**	1207 (3.0)	540 (2.0)	0.0031
**average rank**	261.4	222.9	
**range**	0–23	0–19	
**Category D pDDIs, n (median)**	319 (1.0)	167 (0.0)	0.0143
**average rank**	258.1	228.1	
**range**	0–13	0–7	
**Category X pDDIs, n (median)**	27 (0.0)	25 (0.0)	0.2949
**average rank**	244.0	250.5	
**range**	0–3	0–2	

pDDIs—potential drug–drug interactions.

**Table 6:** Potential DDIs of psycholeptics and antidepressants according to clinical significance and age.

	≥65 Years	<65 Years	*p*
**Patients, n**	302	190	
**pDDIs of psycholeptics and** **antidepressants, n (median)** **average rank** **range**	638 (1.0) 243.2 0–20	450 (1.0) 251.7 0–21	0.5077
**Category C pDDIs of psycholeptics** **and antidepressants, n** **(median)** **average rank** **range**	396 (0.0) 240.2 0–16	310 (1.0) 256.2 0–18	0.1958
**Category D pDDIs of psycholeptics** **and antidepressants, n** **(median)** **average rank** **range**	223 (0.0) 254.6 0–9	127 (0.0) 233.7 0–7	0.0735
**Category X pDDIs of psycholeptics** **and antidepressants, n** **(median)** **average rank** **range**	19 (0.0) 247.4 0–2	13 (0.0) 245.0 0–2	0.6208

pDDIs—potential drug–drug interactions.

**Table 7:** Most common pDDIs of psycholeptics and antidepressants.

Most Common pDDIs of Psychotropic Drugs	n	Potential DDI Consequence
**C interactions**		
**>65 years**		
escitalopram–tramadol	8	Increased risk for serotonin syndrome and seizures.
alprazolam–nitrazepam	8	Increased risk of CNS depression.
diazepam–moxonidine	7	Increased risk of CNS depression.
**<65 years**		
diazepam–promazine	11	Increased risk of CNS depression.
diazepam–mirtazapine	7	Increased risk of CNS depression.
diazepam–valproic acid	7	Increased risk of CNS depression.
**D interactions**		
**>65 years**		
alprazolam–tramadol	25	Increased risk of CNS depression.
oxazepam–tramadol	22	Increased risk of CNS depression.
diazepam–tramadol	20	Increased risk of CNS depression.
**<65 years**		
diazepam–tramadol	20	Increased risk of CNS depression.
diazepam–zolpidem	8	Increased risk of CNS depression.
alprazolam–zolpidem	5	Increased risk of CNS depression.
**X interactions**		
**>65 years**		
promazine–furosemide	8	Diuretics can potentiate the QTc-prolonging impact of promazine.
promazine–indapamide	2	Diuretics can potentiate the QTc-prolonging impact of promazine.
**<65 years**		
diazepam–olanzapine	3	Benzodiazepines can potentiate the adverse effects of olanzapine.
alprazolam–olanzapine	2	Benzodiazepines can potentiate the adverse effects of olanzapine.

pDDIs—potential drug–drug interactions; CNS—central nervous system.

## Data Availability

The data that support the findings of this study are available from the corresponding author upon reasonable request.
